# Supplementary material for: Dynamic transcriptomic profiles of zebrafish gills in response to zinc supplementation
Source: BMC Genomics. 2010 Oct 11;11:553. doi: 10.1186/1471-2164-11-553 (PMC3091702; doi:10.1186/1471-2164-11-553)
Supplement: Additional file 2 — Interactive Direct Interaction Network representing the molecular interactions between zinc, copper, iron, calcium and proteins encoded by transcripts changed by zinc supplementation. Mini web-site containing index.html and hyperlinked pages in subdirectory describing a Direct Interaction Network automatically generated based on curated interactions contained within the proprietary PathwayArchitect database. Ovals represent proteins and the circles symbolize metal ions. Objects are coloured by their abundance in zebrafish at the time-point they were significantly different from the control is a scale from -4 fold (dark green) to +4 fold (dark red). Where significant differences were found at more than one time-point, the colour overlay shows expression at the first instance. Dark blue squares denote 'binding', and light blue squares 'expression'; green squares stand for 'regulation', green diamonds for 'metabolism', and green circles for 'promoter binding'. Arrow heads indicate directionality of the interaction where annotated. All nodes and edges can be further interrogated by selecting the relative area of the image. [file 1471-2164-11-553-S2.zip › PathwayArchitect Zn xs DIN/719202.html]

# REGULATION:

|  |  |
| --- | --- |
| Type | REGULATION |
| Effect | Positive |


---

|  |  |
| --- | --- |
| Score | 0 |


---

|  |  |
| --- | --- |
| Reference Count | 7 |


---

|  |  |
| --- | --- |
| Mechanism | Unknown |


---

|  |  |
| --- | --- |
| Reference:0 || Sentence | "Influence of temperature on relaxation rate as a function of isometric tetanus duration and on Ca2+ and Mg2+ dissociation rates from purified parvalbumin (PA) was examined to test the hypothesis that PA promotes relaxation in frog skeletal muscle." |
| PMID | 1522515 |
| Year | 1992 |
| Journal | J Physiol |
| RefScore | 1 |
| Source | PArchNLP |
  |
|  |  |


---

|  |  |
| --- | --- |
 Reference:1 || Sentence | "The interactions of pike parvalbumins are modulated by Ca2+ and Mg2+ binding to the protein and induce some changes in the physical properties of both the proteins and liposomes." |
| PMID | 3396853 |
| Year | 1988 |
| Species | Human |
| Journal | Gen Physiol Biophys |
| RefScore | 1 |
| Source | PArchNLP |
  ||  |  |


---

|  |  |
| --- | --- |
 Reference:2 || Sentence | "The enthalpy changes determined are distinctly different from those of bullfrog parvalbumins on Ca2+ or Mg2+ binding, but are similar to those on Mg2+-Ca2+ exchange." |
| PMID | 3100327 |
| Year | 1986 |
| Journal | FEBS Lett |
| RefScore | 2 |
| Source | PArchNLP |
  ||  |  |


---

|  |  |
| --- | --- |
 Reference:3 || Sentence | "The rate constant of the conformational change of parvalbumin induced by Ca2+ binding or removal decreases in the order of component 2 greater than component 1 greater than component 5 greater than or equal to component 3; that is, component 2 undergoes the fastest conformational change and component 3 the slowest in response to the rapid free Ca2+ concentration ([Ca2+]) change in the protein solution." |
| PMID | 6438069 |
| Year | 1984 |
| Journal | J Biochem (Tokyo) |
| RefScore | 2 |
| Source | PArchNLP |
  ||  |  |


---

|  |  |
| --- | --- |
 Reference:4 || Sentence | "The possible structural changes in the major isotype of parvalbumin from the toad (Bufo bufo japonicus) skeletal muscle caused by Ca2+ and Mg2+ binding have been analyzed by microcalorimetric titrations." |
| PMID | 2156694 |
| Year | 1990 |
| Journal | Eur J Biochem |
| RefScore | 1 |
| Source | PArchNLP |
  ||  |  |


---

|  |  |
| --- | --- |
 Reference:5 || Sentence | "Steep decreases in rate constant of [Ca(2+)] decay could be explained by an immediate impairment of Ca(2+) uptake and, with longer stimulation periods, by an additional loss of cytosolic Ca(2+) binding capacity resulting from a decay in parvalbumin content." |
| PMID | 10600763 |
| Year | 1999 |
| Species | Rat |
| Journal | Am J Physiol |
| RefScore | 2 |
| Source | PArchNLP |
  ||  |  |


---

|  |  |
| --- | --- |
 Reference:6 || Sentence | "The possible structural changes of the two major isotypes (PA1 and PA2) of parvalbumins from bullfrog (Rana catesbeiana) skeletal muscle caused by Ca2+ binding have been analyzed by microcalorimetric titrations." |
| PMID | 3501318 |
| Year | 1987 |
| Journal | Biochemistry |
| RefScore | 1 |
| Source | PArchNLP |
  |


---

|  |  |
| --- | --- |
